# Dose-Dependent Risk Reduction for Myocardial Infarction with Eicosapentaenoic Acid: a Meta-analysis and Meta-regression Including the STRENGTH Trial

**DOI:** 10.1007/s10557-021-07212-z

**Published:** 2021-06-08

**Authors:** Philip Sarajlic, Gonzalo Artiach, Susanna C. Larsson, Magnus Bäck

**Affiliations:** 1 Department of Medicine Solna, Karolinska Institutet, Stockholm, Sweden; 2 Department of Medicine, Translational Cardiology, Karolinska University Hospital, Hälsovägen 7C, 141 57 Stockholm, Sweden; 3 Unit of Cardiovascular and Nutritional Epidemiology, Institute of Environmental Medicine, Karolinska Institutet, Stockholm, Sweden; 4 Department of Surgical Sciences, Uppsala University, Uppsala, Sweden; 5 Department of Cardiology Huddinge, Karolinska University Hospital, Stockholm, Sweden

**Fig. 1:**
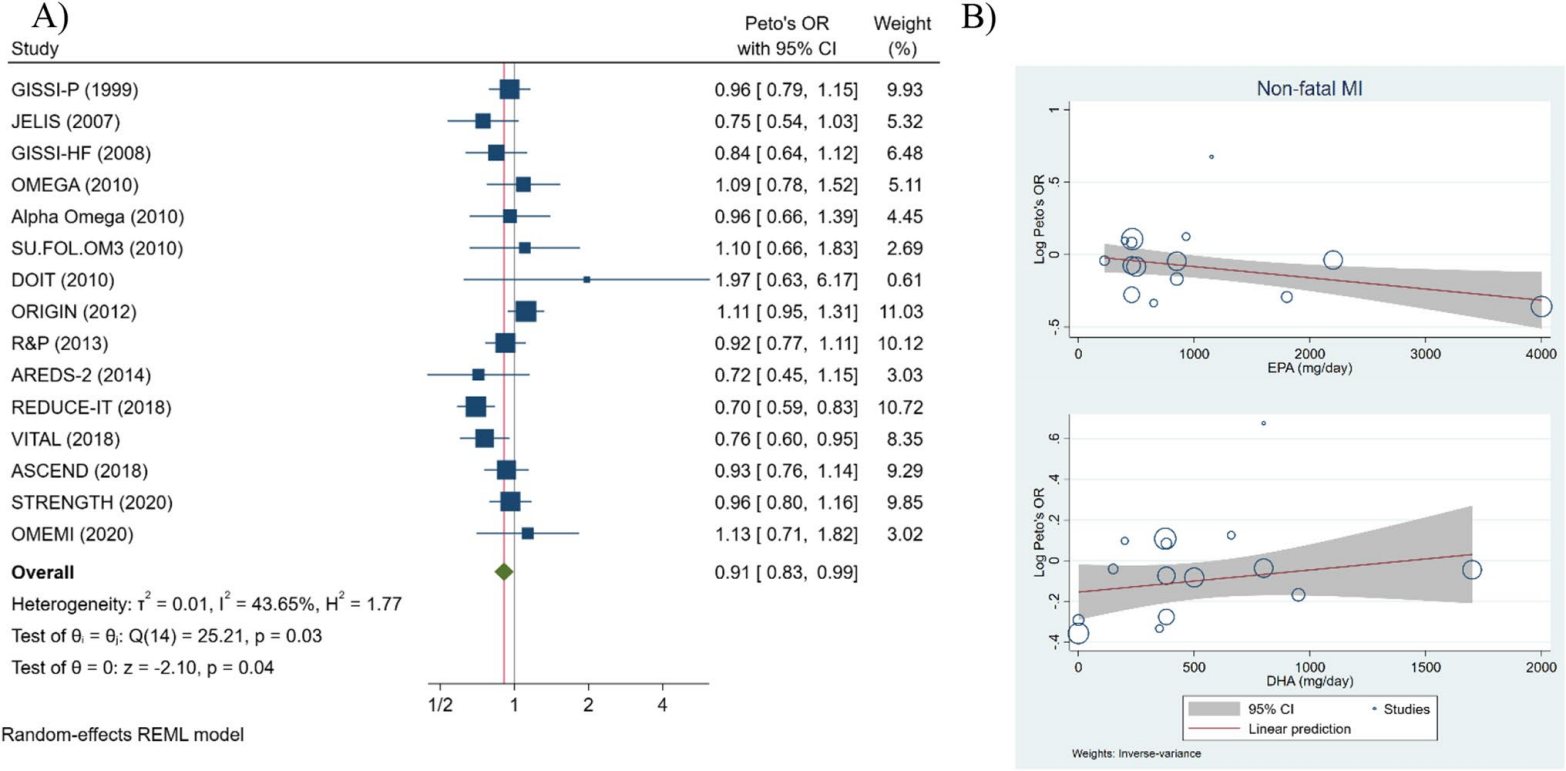
Meta-analysis (**A**) and meta-regression (**B**) with 15 trials illustrating the relationship between omega-3 PUFAs and non-fatal MI risk. (**B**) shows log Peto odds ratios on the y-axis and the omega-3 PUFA dose on the x-axis. Each circle in the scatterplot represents one study, and its area is proportional to the inverse of the standard error

Numerous trials have investigated the role of the long-chain omega-3 polyunsaturated fatty acids (PUFAs) eicosapentaenoic acid (EPA) and docosahexaenoic acid (DHA) for the prevention of cardiovascular events, with renewed interest sparked by recent findings that omega-3 PUFAs are substrates for lipid mediators of the resolution of inflammation [1, 2]. Whereas some meta-analyses indicated no risk reduction for MI by omega-3 PUFA [3], the most recent revealed a significantly 8% lower risk of MI, with higher doses of omega-3 PUFA conferring greatest protection [4]. Since then, the results of the STRENGTH trial were reported and showed that high-dose omega-3 PUFA (4 g, of which 2.2 g EPA and 0.8 g DHA) supplementation had no significant effects on either the composite primary endpoint or non-fatal MI [5]. Therefore, we aimed to update existing meta-analyses [3, 4] with subsequently published trials to determine the association between EPA and DHA and their dosages with MI risk. We included all trials from the meta-analysis by Aung et al. [3] along with five subsequently published trials, namely, REDUCE-IT, VITAL, ASCEND (references in [6]), OMEMI [7], and STRENGTH [5]. The study pool consisted of randomized trials with minimally 500 patients and a follow-up period of at least one year that analyzed the association between omega-3 PUFA supplementation and vascular events. In total, 15 relevant trials were included. While both fatal and non-fatal MI outcomes were analyzed, in this report, we present analyses on non-fatal MI risk since more non-fatal MI events were recorded in the included trials, making this approach more statistically powerful. For each trial, Peto odds ratio was calculated to determine effect sizes. A meta-analytic scatterplot was created to visualize the risk of MI in each trial based on the dosage for EPA and DHA, respectively, using a random-effects model. A meta-analytic regression line was fitted in the scatterplot to determine the risk trend and slope for the two omega-3 PUFAs. All statistical calculations were done using the suite of commands, “meta,” in Stata version 16 (StataCorp. 2019. *Release 16*. College Station, TX: StataCorp LLC). A two-sided alpha value of 0.05 was used to determine statistical significance. Despite the non-significant effects of the latest trial [5], omega-3 supplementation was associated with a statistically significant lower odds of non-fatal MI (odds ratio 0.91; 95% CI 0.83–0.99) in the meta-analysis of 15 studies, with moderate heterogeneity between estimates from individual trials (I^2^ = 44%) (Fig. 1A). A significant dose-dependent risk reduction of non-fatal MI was observed for EPA (Fig. 1B). While DHA was significantly associated with a lower risk of non-fatal MI at low doses, the risk reduction lost significance at higher doses (Fig. 1B). In a bivariate meta-regression analysis, with EPA and DHA as covariates, EPA achieved a significant non-fatal MI risk reduction (*P* = 0.048; z = -1.97), while the effect of DHA was non-significant (*P* = 0.477; z = 0.71). In a sensitivity analysis including only double-blind trials, a univariate meta-regression revealed significant (*P* = 0.048; z = -1.98) beneficial risk reduction properties of EPA. Together, these findings point to a dose-dependent risk reduction of non-fatal MI with increasing EPA dosage, regardless of DHA intake. In order to account for these differential effects, one could look at atherosclerosis and its pathophysiology. In addition to their anti-thrombotic, triglyceride-lowering, and atherogenic remnant particle lowering effects, EPA and DHA serve as substrates for specialized pro-resolving mediators (SPMs)^2^, which promote the resolution of atherosclerotic inflammation [1]. Preclinical atherosclerosis models indicate that EPA leads to the formation of SPMs capable of tipping the cardiovascular homeostatic balance towards inflammation resolution [8]. A limitation of our meta-analysis is the presence of variances in disease severity across different study populations, potentially contributing to heterogeneity between the trials. Furthermore, analyses on fatal MI were not feasible due to a lack of reported outcome data in the included trials. In conclusion, this contemporary meta-analysis showed that EPA was associated with a significant risk reduction of non-fatal MI in a dose-dependent fashion. The association persisted in a model adjusting for DHA intake, emphasizing the role of EPA supplementation in CHD prevention. Further studies on EPA downstream metabolites are warranted.
